# Metabolic Adaptations in Cancer Progression: Optimization Strategies and Therapeutic Targets

**DOI:** 10.3390/cancers17142341

**Published:** 2025-07-15

**Authors:** Agnieszka Dominiak, Beata Chełstowska, Grażyna Nowicka

**Affiliations:** 1 Department of Biochemistry and Pharmacogenomics, Medical University of Warsaw, 02-097 Warsaw, Poland; grazyna.nowicka@wum.edu.pl; 2 Centre for Preclinical Research, Medical University of Warsaw, 02-097 Warsaw, Poland; 3 Department of Biochemistry and Laboratory Diagnostics, Faculty of Medicine, Collegium Medicum, Cardinal Stefan Wyszyński University, 01-938 Warsaw, Poland; b.chelstowska@uksw.edu.pl

**Keywords:** amino acids utilization, anticancer metabolic strategies, dietary interventions, fatty acid synthesis, glucose metabolism, gut microbiota, metabolic reprogramming, precision oncology, tumor metabolism, tumor microenvironment

## Abstract

As tumor research has deepened, the deregulation of cellular metabolism has emerged as yet another recognized hallmark of cancer. Tumor cells adapt different biochemical pathways to support their rapid growth, proliferation, and invasion, resulting in distinct anabolic and catabolic activities compared with healthy tissues. Certain metabolic shifts, such as altered glucose and glutamine utilization and increased de novo fatty acid synthesis, are critical early on, while others may become essential only during metastasis. These metabolic adaptations are closely shaped by, and in turn remodel, the tumor microenvironment, creating favorable conditions for their spread. Anticancer metabolic strategies should integrate pharmacological approaches aimed at inhibiting specific biochemical pathways with well-defined dietary interventions as adjunctive therapies, considering also the role of gut microbiota in modulating diet and treatment responses. Given the established link between the consumption of foods rich in saturated fatty acids and sugars and an increased cancer risk, the effects of diet cannot be ignored. However, current evidence from controlled and multicenter clinical trials remains insufficient to provide definitive clinical recommendations. Further research using modern omics methods, such as metabolomics, proteomics, and lipidomics, is necessary to understand the changes in the metabolic profiles of various cancers at different stages of their development and to determine the potential for modifying these profiles through pharmacological agents and dietary modifications. Therefore, clinical trials should combine standard treatments with novel approaches targeting metabolic reprogramming, such as inhibition of specific enzymes and transporters or binding proteins, alongside the implementation of dietary restrictions that limit nutrient availability for tumor growth. However, to optimize therapeutic efficacy, a precision medicine approach should be adopted that balances the destruction of cancer cells with the protection of healthy ones. This approach, among others, should be based on cell type-specific metabolic profiling, which is crucial for personalizing oncology treatment.

## Simple Summary

Tumor development requires cancer cells to increase their energy production. They need this energy to synthesize a variety of macromolecules that support the formation and development of new tumor cells. These cells also interact with their environment to create favorable conditions for malignancy. Developing effective methods for early diagnosis, treatment, and monitoring of cancer requires a thorough understanding of the metabolic changes that accompany its initiation and progression. Nowadays, it is believed that we have to develop precise principles for complex anticancer therapies. These should be supported by new drugs that disrupt the metabolism of cancer cells and specific dietary interventions that reduce their metabolic efficiency. In this article, we discuss recent discoveries in the field of energy metabolism in cancer cells that drive the proliferation of metastatic tumors and suggest ways to improve cancer treatment. Analyzing each tumor’s metabolism individually can help create personalized therapies that kill cancer cells while protecting healthy tissue.

## 1. Introduction

Cancer, as a disease, has been known for millennia, and despite the advances in modern medicine, it remains one of the leading causes of death worldwide. Excessive cell proliferation and invasion, connected with the decline of cellular control mechanisms, are hallmarks of all cancers. In addition, tumor cells adapt biochemical pathways to survive; thus, their anabolism and catabolism differ from the metabolism of normal, healthy tissue. The biochemical processes of cancer progression have long been the subject of intense research. Yet, there remains a need to deeply explore the changes in cell metabolism during cancer evolution to find a way to develop effective anticancer therapies by interfering with the metabolism of cancer cells, resulting in reducing tumor growth and limiting the emergence of treatment resistance.

Under favorable conditions, most primary cancer cells—those at the site where the cancer originated—circulate in the bloodstream and migrate throughout the body, colonizing distant sites and causing symptoms depending on where they have spread. Metastasis is an evolutionary event in cancer progression. It has long been accepted that malignant tumors exhibit organ-specific metastasis patterns, which align with Paget’s “seed and soil” theory [1]. By way of illustration, colon carcinomas spread more often into the liver and lungs but less frequently to the brain, bone, or skin, whereas breast cancers tend to metastasize to the bones, lungs, brain, or liver.

The primary tumor is, in most cases, susceptible to eradication using classical surgery and radio- and chemotherapy or modern immunotherapies such as checkpoint inhibitors and chimeric antigen receptor T cells. The efficacy of intervention largely depends on sufficiently early diagnosis, whereas metastasis makes treatment more difficult and finally causes the patient’s death. Thus, most cancers can be cured if diagnosed before cells have spread outside the tissue of origin. The metabolic changes associated with tumor spread are thought to be one of the key causes of cancer treatment failure. Intensive energy generation, increased macromolecular biosynthesis, and specific signal transmission are well-known characteristics of all cancer cells [2]. To avoid nutrient limitation, some altered metabolic processes, particularly aerobic glycolysis, amino acid (a.a.) utilization, and lipid uptake in tumors, become essential very early, while others are dispensable in primary tumors but obligatory in metastasis. It has been observed that glucose uptake in cancer cells is 10 times more extensive in comparison with normal tissue. This discovery of increased glucose uptake has provided the basis for the clinical application of fluorodeoxyglucose positron emission tomography (FDG-PET) imaging, which is currently routinely used for cancer detection and monitoring. Additionally, glutamine (Gln), glycine, and aspartate are necessary for purine and pyrimidine synthesis, while serine (Ser) and essential amino acids (e.a.a.) are vital for the synthesis of membrane lipid components. In summary, tumor cells actively acquire energy sources through the conversion of glucose, fatty acids, and a.a.s. to produce the biomolecules necessary for their growth. Therefore, it seems reasonable to develop principles for the implementation of dietary interventions with the simultaneous inhibition of specific biochemical pathways as an additional component for cancer treatment. However, rigorous clinical validations are needed to translate such adjuvant therapy from the bench to the bedside. This is particularly important given the complexity of the tumor microenvironment and the close interactions between cancer cells and surrounding stromal components. For example, stromal cancer-associated fibroblasts (CAFs) notably enhance Gln synthesis and secrete it to support and promote cancer cell growth [3].

The field of cancer metabolism has evolved from the primary model of the Warburg Effect—where cancer cells preferentially rely on aerobic glycolysis, converting glucose to lactate even in the presence of oxygen—in the first half of the twentieth century to our current understanding of the complexity and flexibility of the metabolic network in tumors. Visualizing the dynamics in the metabolic switch during cancer initiation and development will help to identify targets for diagnosis, monitoring, and optimized specific therapeutic intervention. However, metabolic regulators have not yet been successfully translated into clinical practice [4], and targeting cancer metabolism remains a promising strategy for cancer therapy. Cellular metabolism research often relies on stable isotopes, such as 13C-labeled or deuterated metabolites, to gain insights into the activity of metabolic pathways. However, this methodology is not typically used in routine clinical practice. In the clinic, it would be possible to assess specific metabolites because such a methodology already exists and can be adapted to clinical practice needs and used in therapy programming.

Proposing an evaluation of characteristic metabolites requires a deep understanding of the changes in the metabolic profile of cancer cells at every stage of their development. Various aspects of cancer metabolism reprogramming have been reviewed recently [5,6,7,8]. Here, we discuss the specific discoveries in the field of energy metabolism in cancer cells that fuel the proliferation of metastatic tumors and offer suggestions for improving therapeutic approaches. However, the task remains complex because different cancer types have unique metabolic phenotypes and requirements.

## 2. Glucose Metabolism Reprogramming in Cancer

In normal cells, glucose is metabolized through glycolysis to pyruvate, which is then mostly directed into the tricarboxylic acid cycle (TCA) for oxidative phosphorylation, yielding ATP. However, in cancer cells, pyruvate is predominantly converted to lactate by lactate dehydrogenase A (LDHA), facilitating rapid ATP production and regeneration of NAD^+^, essential for sustaining high glycolytic flux. This metabolic reprogramming supports the biosynthetic and energetic demands of proliferating tumor cells [9]. The extent of aerobic glycolysis varies between tumor types and can also differ within a single tumor. The increased expression of genes involved in oxidative phosphorylation was shown in ovarian, lymphoma, leukemia, and lung cancers, while thyroid, colon, pancreatic, and renal cancers showed the opposite pattern [10].

A hallmark of cancer adaptations is the reprogramming of glucose metabolism, characterized by increased glycolysis and subsequent lactate production, even in the presence of oxygen. This metabolic shift provides energy and biosynthetic precursors and affects the tumor microenvironment. Lactate, once considered a mere byproduct, is now recognized as a central metabolite in tumor biology. It serves as a fuel for oxidative metabolism in both cancer and stromal cells, modulates the immune response, and contributes to angiogenesis and metastasis. The export and import of lactate are mediated by monocarboxylate transporters (MCTs), particularly MCT1 and MCT4, which maintain intracellular pH and facilitate metabolic crosstalk within the tumor microenvironment [9,11]. The secretion of large amounts of lactic acid creates a hypoglycemic and acidic tumor microenvironment. This low pH disrupts the function of mesenchymal cells within the tumor, particularly immune cells. Additionally, lactic acid promotes the polarization of tumor-associated macrophages toward the M2 phenotype, driving malignant tumor progression via the lactate–MCT HIF1α signaling pathway. Hypoxia, a hallmark of solid tumors, further exacerbates this microenvironment by stabilizing hypoxia-inducible factor 1 alpha (HIF1α), which enhances lactate production, thereby promoting tumor survival and aggressiveness [12]. Lactate accumulation in the tumor microenvironment has been implicated in various processes that facilitate metastasis:

- Immune Evasion: Elevated lactate levels suppress the function of immune cells, including T lymphocytes and natural killer cells, enabling tumor cells to evade immune surveillance [13].
- Angiogenesis: Lactate acts as a signaling molecule, inducing the expression of vascular endothelial growth factor (VEGF) and promoting the formation of new blood vessels, which supply nutrients and oxygen to metastatic tumors [14].
- Cell Migration and Invasion: Lactate influences the expression of matrix metalloproteinases (MMPs) and hyaluronan production, remodeling the extracellular matrix and facilitating cancer cell migration and invasion [15].
- Alternative Energy Source: Lactate can serve as an energy source for both tumor cells and endothelial cells, supporting the energy demands of metastatic growth [16].

Moreover, cancer-associated fibroblasts in the tumor stroma undergo aerobic glycolysis, producing lactate that is utilized by adjacent cancer cells for oxidative metabolism in a process called the Reverse Warburg Effect. This metabolic coupling supports tumor growth and survival in metastatic sites [17].

**Figure 1:**
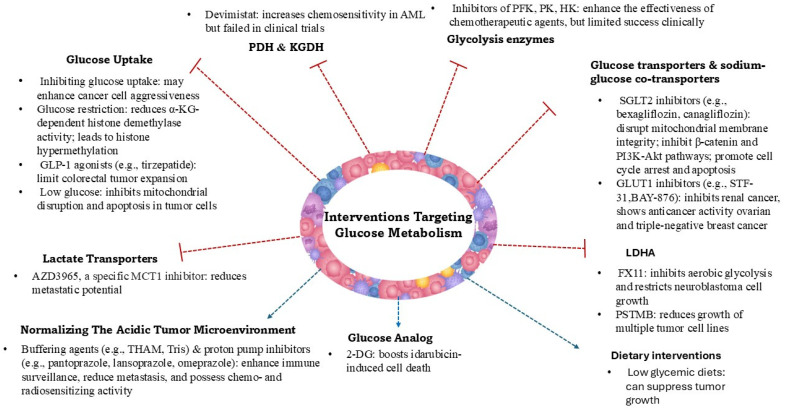
Summary of various glucose-targeted metabolic therapeutic approaches in the fight against cancer.

Since cancer cells rely on the aerobic glycolytic pathway for ATP production and have a higher demand for glucose, targeting cellular glucose uptake and glycolysis seems an appealing way to fight cancer. Several glycolytic enzymes, including phosphofructokinase, pyruvate kinase, and hexokinase, as well as members of the glucose transporter (GLUT) and Na^+^/glucose co-transporter (SGLT) families, are overexpressed or overproduced in cancer cells (such as those in breast, lung, and ovarian cancers) and have been analyzed in experimental and some human studies as targets for cancer therapy (Figure 1). SGLT2 inhibitors fight cancer by altering metabolism, reducing inflammation, and lowering oxidative stress [18,19,20,21].

Another approach involves the use of 2-deoxy-D-glucose (2-DG), a glucose analog that is phosphorylated by hexokinase into 2-deoxy-D-glucose-6-phosphate but is not further processed or accumulated within the cells. It was shown that 2-DG boosts idarubicin-induced cell death in P388 leukemia cells [22,23]. 2-Deoxy-D-glucose alone or combined with docetaxel was studied in a phase I clinical trial in patients with advanced solid tumors (NCT00096707) [24]. However, this solution has not been translated into clinical practice. Yet, radioisotope analogs of deoxy-glucose as ^18^F-2-fluoro-2-deoxy glucose (^18^F-FDG) are radiotracers widely used in positron emission tomography (PET) to detect transformed cells, as ^18^F-FDG is converted to ^18^F-FDG-6-phosphate and accumulates in cells that intensively metabolize glucose [25].

Generally, inhibiting glycolysis on its own has proven to be ineffective in clinical practice [26]. It is necessary to emphasize that glycolysis is crucial to normal cell metabolism. In addition, cancer cells are highly flexible and adaptable, and they use a.a.s. and fatty acids to produce energy when deprived of glucose [27]. However, the inhibition of glycolytic enzymes such as hexokinase, pyruvate kinase, and lactate LDHA has been shown to enhance the effectiveness of several chemotherapeutic agents [28]. Data from studies on lung adenocarcinoma and triple-negative breast cancer have shown that glycolytic cancer cell phenotypes are generally associated with worse patient survival [29,30,31]. Type 2 diabetes mellitus (T2D) has been recognized as a cancer risk factor, with an increased incidence of colorectal and ovarian cancers observed in T2D patients. Moreover, antidiabetic treatments such as tirzepatide have been recognized to affect anticancer therapy [32,33]. However, the effects of an antidiabetic regimen on cancer risk and outcomes are not clear, as indicated by a recent study [34]. In addition, it has been shown that the high glucose requirement by cancer cells causes the tumor microenvironment to become low in glucose. This, in turn, inhibits the consumption of uridine nucleotide resources by tumor cells, which in turn reduces the effectiveness of chemotherapy aimed at inhibiting nucleotide and nucleic acid biosynthesis. At the same time, the low-glucose tumor microenvironment prevents the activation of Bcl-2-associated X protein (BAX) and Bcl-2 homologous antagonist/killer (BAK) proteins (present on the surface of mitochondria) in cancer cells, thus inhibiting mitochondrial disruption and apoptosis [35]. It was also recognized that inhibiting cellular glucose uptake and glycolysis can enhance the aggressiveness of malignant cells, while at the same time, insufficient glucose in the tumor microenvironment has been shown to reduce the effectiveness of T cell antitumor responses. Glucose restriction led to a reduction in alpha-ketoglutarate (α-KG) levels and decreased the activity of α-KG-dependent histone demethylases, resulting in histone hypermethylation. These epigenetic changes increased the expression of HIF1α and HIF1α-dependent gene transcription, promoting the dedifferentiation of malignant cells into a more aggressive phenotype [36,37,38]. The results of the presented studies indicate that understanding not only the reprogramming of key metabolic processes in cancer cells, but also the associated epigenetic modifications and changes in the tumor microenvironment, is essential for developing effective therapies and minimizing the risk of resistance to treatment.

Emerging evidence suggests that metastatic cells undergo further metabolic reprogramming to adapt to the distinct microenvironments of metastatic sites. During metastasis, cancer cells often shift from a proliferative to a migratory phenotype, necessitating metabolic adaptations. Anaplerotic pathways, such as the pyruvate carboxylase (PC)-mediated conversion of pyruvate to oxaloacetate, replenish TCA cycle intermediates, supporting the energy and biosynthetic needs of migrating cells. In breast cancer, increased PC activity has been linked to enhanced invasiveness and metastatic potential, although the precise mechanisms remain to be elucidated. Additionally, pyruvate oxidation via pyruvate dehydrogenase (PDH) produces acetyl-CoA, which can influence gene expression through histone acetylation. Inhibition of acetyl-CoA-consuming enzymes, such as acetyl-CoA carboxylase 1 (ACC1), leads to acetylation of transcription factors like Smad2, promoting mesenchymal gene expression patterns associated with metastasis.

Pyruvate dehydrogenase (PDH) and α-ketoglutarate dehydrogenase (KGDH) are essential entry points for monosaccharides and a.a.s. into the Krebs cycle and are therefore integral to mitochondrial bioenergetics. Devimistat (CPI-613), a lipoic acid antagonist that selectively inhibits PDH and KGDH, was designed to disrupt energy production in cancer cells. Devimistat was recognized to increase sensitivity to chemotherapy in acute myeloid leukemia (AML) cells and in a group of older patients with relapsed or refractory AML treated with cytarabine and mitoxantrone [39]. However, the phase III multicenter ARMADA 2000 trial (NCT03504410) showed that devimistat added to chemotherapy did not improve remission rates or overall survival in AML patients [40]. Also, the combination of devimistat with fluorouracil, oxaliplatin, irinotecan, and leucovorin did not improve short- or long-term outcomes in patients with metastatic pancreatic adenocarcinoma as shown by the phase III AVENGER 500 trial (NCT03504423) [41]. The high metabolic plasticity of cancer cells, which contributes to their aggressive behavior, is undoubtedly a key reason for the lack of clinical benefit of the presented therapeutic strategies. This ability of the tumor to adapt may result from the presence of multiple clones with different metabolic features, wherein treatment acts like natural selection, favoring the survival of the most resistant cells. Therefore, a deeper understanding of the ability of cancer cells to use different salvage pathways to alter their metabolism to enable survival, development, and metastasis is needed.

Metastatic tumors often exhibit metabolic symbiosis, where subsets of cancer cells adapt their metabolism to the microenvironmental conditions of the metastatic sites. Hypoxic tumor regions produce and secrete lactate via MCT4, which is taken up by oxygenated cancer cells through MCT1 and utilized for oxidative phosphorylation. This lactate shuttle supports metabolic flexibility and efficient energy production within the tumor. Possible treatment options are related to the inhibition of lactate transporters. Pharmacological blockade of MCT1 and MCT4 disrupts lactate transport, resulting in intracellular acidification and decreased metastatic potential. Clinical trials are currently underway to assess the efficacy of MCT inhibitors across various cancer types [42,43].

It is also known that inhibitors of LDHA such as oxamate, aminooxyacetate, and dichloroacetate reduce lactate production, thereby decreasing the invasion and migration capabilities of cancer cells. Preclinical studies have demonstrated the potential of LDHA inhibitors in suppressing metastasis, with notable examples including FX11, which effectively blocks aerobic glycolysis and restricts the growth of neuroblastoma cell lines [44]. Another promising inhibitor, 1-(phenylseleno)-4-(trifluoromethyl) benzene (PSTMB), has demonstrated the ability to decrease the growth of multiple tumor cell lines, including NCI-H460 (human non-small-cell lung carcinoma), MCF-7 (human breast cancer), Hep3B (human hepatocellular carcinoma), A375 (human malignant melanoma), HT29 (human colorectal adenocarcinoma), and LLC (murine lung carcinoma) [45].

Strategies aimed at normalizing the acidic tumor microenvironment, such as buffering agents (e.g., tris(hydroxymethyl)aminomethane) or inhibitors of proton pumps, may enhance immune surveillance and reduce metastatic spread. One of the earliest therapeutic approaches to alter lactic acid metabolism was the neutralization of lactic acid in vivo using an alkaline buffer, though this method has proven challenging to apply in clinical practice [46,47].

The reprogramming of pyruvate and lactate metabolism plays a critical role in the progression and metastasis of cancer. While primary tumors rely heavily on glycolysis and lactate production to sustain rapid proliferation, metastatic tumors develop additional metabolic adaptations to thrive in diverse microenvironments. Understanding these metabolic differences provides valuable insights for the development of novel therapeutic strategies aimed at targeting metabolic vulnerabilities in cancer [9].

## 3. Fatty Acid Metabolism in Cancer

Both glucose and fatty acid (FA) reprogramming are actively being investigated, with the advancement of omics technologies such as lipidomics and genomics playing a crucial role in enhancing our understanding of the changes in lipid metabolism associated with cancer development [48]. Cancer cells grow and develop in an environment of low oxygen, nutrient deprivation, and oxidative stress, and their activity is inextricably linked to lipid metabolism. The manner in which cancer cells metabolize lipids is frequently shaped by the intricate dynamics of the tumor microenvironment. Adipocytes can be triggered by cancer cells to break down their triglyceride reserves, releasing FAs that are then taken up by cancer cells. Additionally, CAFs contribute to lipid secretion, which supports cancer cell metabolism [49]. To meet their energy demands, cancer cells uptake lipids through the action of transporter and translocase proteins.

**Figure 2:**
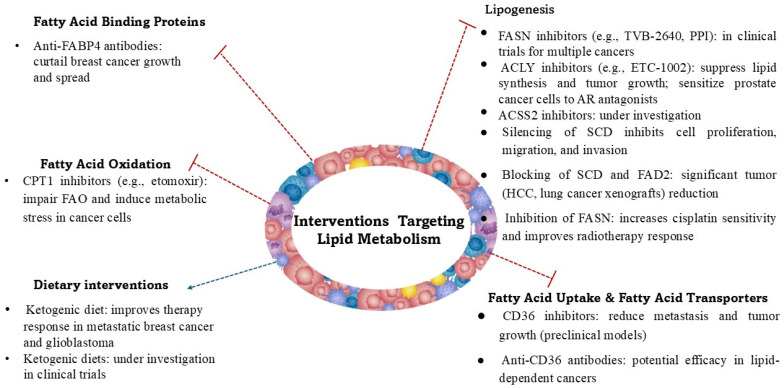
Summary of various lipid-targeted metabolic therapeutic approaches in the fight against cancer.

Fatty acids function not only as an energy source, but also as essential structural components of cellular membranes and as signaling molecules that influence various oncogenic pathways. Via FAs, cholesterol, and phospholipids, cancer cells bidirectionally communicate with immune and stromal cells, the main components of the TME [50]. Therefore, recognition of the metabolic differences between primary and metastatic tumors as well as cancers cells’ interactions with the microenvironment in terms of fatty acid synthesis, uptake, and oxidation is necessary to provide potential new therapeutic targets (Figure 2) for cancer treatment. Fatty acid metabolism and its reprogramming are actively being investigated, with the advancement of omics technologies such as lipidomics and genomics playing a crucial role in enhancing our understanding of the changes in lipid metabolism associated with cancer development.

Unlike normal cells, which mostly rely on dietary lipids, cancer cells (such as those in breast, prostate, colorectal, and hepatocellular carcinomas) often exhibit increased de novo FA synthesis [51,52]. This process is driven by the upregulation of key lipogenic enzymes, including fatty acid synthase (FASN), which catalyzes the synthesis of palmitate from acetyl-CoA and malonyl-CoA. Overexpression of FASN is linked to tumor progression and poor prognosis in several cancers, including triple-negative breast cancer, where it is upregulated in 70% of newly diagnosed cases [53]. In cancer cells, FASN overexpression promotes their survival and proliferation via the activation of HER1/2 and EGFR receptors, which in turn induce FASN expression via the activation of the MAPK and PI3K/Akt/mTOR pathways [54]. The second upregulated enzyme is ATP-citrate lyase (ACLY), which converts citrate into acetyl-CoA, a precursor for FA synthesis, and the third one is acetyl-CoA carboxylase (ACC), converting acetyl-CoA into malonyl-CoA, the first committed step in FA biosynthesis. One of the pioneering studies highlighting the significance of lipid metabolism in cancer showed that ACLY activity was 160 times higher in breast cancer tumors compared with normal tissue [55]. Additionally, elevated levels of ACLY activity are linked to poor prognosis in various tumor types [56]. In addition, under conditions of metabolic stress such as hypoxia or lipid depletion, cancer cells upregulate acetyl-CoA synthetase 2 (ACSS2) to generate acetyl-CoA from acetate [57]. Targeting FA synthesis by inhibiting FASN, ACLY, and ACSS2 has been the subject of numerous preclinical and several clinical studies in various cancers, including breast, lung, colon, prostate, and bladder [58]. Fatty acid synthesis is closely associated with the activation of fatty acid desaturation and elongation. Stearoyl-CoA desaturase (SCD) converts saturated FAs into monounsaturated fatty acids (MUFAs), thereby enhancing lipid utilization within the tumor microenvironment. Silencing of SCD was found to inhibit cell proliferation, migration, and invasion [59]. Additionally, cancer cells also use an alternative desaturation pathway involving the FADS2 (fatty acid desaturase 2)-dependent desaturation of palmitate to sapienate (*cis*-6-C16:1) to support the synthesis of specific lipids and cellular membranes during proliferation. FADS2 has been shown to play a key role in FA desaturation in different cancer cell lines and primary tumors that are resistant to SCD inhibitors, and simultaneous blocking of both SCD and FADS2 resulted in a significant tumor reduction in hepatocellular and lung carcinoma xenografts [60]. Increased lipogenesis provides cancer cells with the essential membrane components required for proliferation and adaptation to stressful conditions. Moreover, lipogenesis is driven by oncogenic signaling and, reciprocally, lipogenic pathways contribute to oncogenic signaling, including the PI3K-AKT-mTOR and MYC pathways [61].

Apart from endogenous synthesis, cancer cells can scavenge exogenous FAs from the tumor microenvironment. This uptake is facilitated by the increased activity of fatty acid transport proteins (FATPs), which mediate the transport of long-chain fatty acids. Enhanced expression of the CD36 protein, which acts as both a signaling receptor and a fatty acid transporter (known as fatty acid translocase), has been recognized particularly in various metastatic tumors. CD36 facilitates cellular FA uptake, leading to increased FA oxidation and providing cells with energy, promoting their proliferation and metastasis [62,63]. In tumors, CD36 is expressed not only by cancer cells but also by stromal cells and immune-infiltrating cells. It influences immune cell recruitment and participates in creating an immunosuppressive environment and resistance to anticancer therapy [64]. High CD36 expression is linked to increased invasiveness and metastatic potential in several cancers, including breast, ovarian, prostate, and colorectal cancers. CD36 is a potential target for anticancer therapy. Its function can be inhibited using specific monoclonal antibodies and CD36 antagonists, the antitumor and antimetastatic effectiveness of which is indicated by in vitro and animal studies [65,66].

In tumor cells, enhanced expression of fatty acid binding proteins (FABPs) is also observed, and their expression is stimulated by HIF1α [67]. Increased FABP expression in adipocytes and macrophages within the tumor microenvironment is an important factor influencing cell–cell interactions, fatty acid accumulation in cancer cells, and cancer progression, as demonstrated in the context of breast cancer [68]. Since FABPs facilitate lipid transport and metabolism, the expression patterns of FABP4 and FABP5 in breast cancer have emerged as potential prognostic markers. Moreover, inhibition of FABP4 and/or FABP5 is being explored as a novel therapeutic strategy [69]. Cancer cells store excess FAs in lipid droplets, which protect them from lipotoxicity, serve as an energy reservoir during metabolic stress, and supply NADPH, which plays a key role in protecting cancer cells against ROS-induced toxicity [61,70].

Fatty acids are a key substrate for energy production, and fatty acid oxidation (FAO), known as β-oxidation, provides ATP and NADPH to support cancer cell development and survival under metabolic stress. In cancer cells, key upregulated enzymes involved in FAO include the following:

- Carnitine palmitoyltransferase 1 (CPT1) regulates the entry of FAs into mitochondria for oxidation. The overexpression of CPT1A has been observed in various cancers and is linked to resistance to metabolic stress [71].
- Acyl-CoA dehydrogenases are responsible for catalysis of the initial step in FA oxidation [72].
- Peroxisome proliferator-activated receptor (PPAR) signaling regulates FAO and lipid metabolism in cancer cells [73].

FAO is particularly important in metastatic cancer cells, such as those in triple-negative breast cancer, which rely on FAs as an alternative energy source when glucose availability is limited. Studies suggest that metastatic tumors, especially in lipid-rich environments such as the bone marrow or adipose tissue, exhibit enhanced FAO to support their survival and proliferation [61,74].

Primary tumors (breast, prostate, colorectal, ovary, and upper gastrointestinal tract cancers) often exhibit high rates of de novo FA synthesis, whereas metastatic tumors (lung, breast, and ovarian cancers) shift toward increased FA uptake and oxidation to adapt to their new microenvironments [75,76,77]. It is worth noting that the primary microenvironment of one tumor type may serve as the metastatic niche for another. Both hypoxia and an acidic microenvironment drive the reprogramming of FA metabolism in cancer cells through changes in enzyme activity, mitochondrial and histone acetylation, and gene expression [48,78]. Oncogenic signaling also plays an important role in metabolic reprogramming. Increased fatty acid uptake occurs under normoxic conditions following oncogenic Ras activation, accompanied by reduced oxygen consumption, elevated citrate synthesis from reductive carboxylation (which enables cells to convert glutamine-derived α-KG to citrate), and consequent independence from SCD-1 for producing unsaturated fatty acids [79]. Activation of saturated and monounsaturated FA synthesis affects the FA composition of cellular membranes as well as membrane permeability and potential, which in turn are associated with resistance to ROS-induced apoptosis and cancer progression [80].

Lipid metabolism reprogramming is involved not only in cancer development, but also in treatment resistance. Increased de novo lipogenesis results in saturated fatty acids’ incorporation into cell membranes, which enhances cell resistance to chemotherapy. FAO activation provides ATP for drug efflux mechanisms and NADPH for reducing oxidative stress and promoting cell survival. Similarly, increased cholesterol uptake and synthesis in cancer—driven by its structural role in membrane lipid rafts—contribute to tumor progression by enhancing signal transduction pathways. The widespread clinical use of statins, drugs that modulate cholesterol metabolism by inhibiting HMG-CoA (3-hydroxy-3-methylglutaryl-coenzyme A) reductase, has significantly reduced the incidence and mortality of cardiovascular diseases. Notably, dyslipidemia has also been identified as an important risk factor for cancer initiation, progression, and metastasis. Additionally, bile acids produced through cholesterol transformation exert a direct influence on the gut microbiota, which in turn is associated with cancer development and prognosis. In this context, preclinical studies have demonstrated that statins possess both preventive and therapeutic potential across various cancer types, including the ability to enhance the efficacy of anticancer treatments. Nevertheless, despite these promising observations, the role of statins in oncology remains a subject of ongoing debate. Many clinical trials (NCT00811087; NCT00295514; NCT00360025) do not demonstrate a significant benefit of statins in cancer treatment [81,82,83]. However, modulating the lipid signaling pathways involved in apoptosis resistance and targeting lipid metabolism could improve the efficacy of treatments and overcome resistance in aggressive cancers. For example, inhibition of FASN has been shown to improve cisplatin sensitivity in chemoresistant ovarian cancer cells [84]. It also increases cancer cell sensitivity to radiotherapy [85]. Proton pump inhibitors (PPIs) have been shown to specifically suppress FASN activity and trigger apoptosis in breast cancer cell lines while having little impact on non-cancerous cells [86]. To assess the clinical usefulness of FASN inhibition, the phase I study (NCT02223247) on TVB-2640 (denifanstat), an FASN inhibitor, was conducted in patients with advanced solid malignant tumors. TVB-2640 has also been used in a prospective, single-arm, two-stage phase II clinical trial (NCT03808558) in non-small-cell lung cancer patients with KRAS-mutant, in colorectal cancer (NCT02980029), HER2^+^ advanced-stage breast cancer (NCT03179904), high-grade astrocytoma (NCT03032484), and steatohepatitis (NCT03938246), and the results of these clinical trials are pending.

Research has also focused on ACLY inhibition. Bempedoic acid (ETC-1002), an ACLY inhibitor, was found to induce apoptosis and inhibit cancer cell invasion, and in combination with palbociclib (a CDK4/6 inhibitor), it significantly reduced cell viability in a panel of breast and pancreatic cancer cell lines [87]. ALCY inhibition also sensitized prostate cancer cells to androgen receptor antagonism [88].

Lipid reprogramming associated with cancer development involves not only cancer cells but also cells within the tumor microenvironment, including immune cells. Escape from immune surveillance is an important element that enables cancer to develop. Recent studies suggest that targeting lipid metabolism can enhance the efficacy of immunotherapies. By modulating the lipid metabolic pathways within the tumor microenvironment, it is possible to improve immune cell function, particularly of T cells and dendritic cells, thereby promoting a more robust antitumor immune response [89].

The functioning of the human body depends on the supply of nutrients from the diet, including FAs. The amount and type of FAs consumed significantly affect metabolism and the risk of diseases, including cancer. It is therefore difficult to ignore the effects of dietary FAs when discussing the changes in FA metabolism associated with cancer development and their implications for cancer treatment. A high intake of fats rich in saturated fatty acids has been related to increased cancer risk, particularly affecting breast, prostate, and colorectal cancers [90]. As summarized in the recent review [91], polyunsaturated fatty acids (PUFAs) affect tumor cell proliferation; cell death, migration, and invasion; energy metabolism remodeling; epigenetics; and immunity. Omega-3 PUFAs inhibit cell proliferation, promote cancer cell death, suppress cancer metastasis, alter energy metabolism, and inhibit tumor microenvironment inflammation. However, omega-6 PUFAs exhibit weaker antitumor effects and may promote tumor development, supporting an inflammatory tumor microenvironment. Epidemiological studies indicate that a diet with high n-6/n-3 PUFA increased cancer risk [92]. In clinical studies, supplementation with n-3 PUFAs such as eicosapentaenoic acid (EPA) and docosahexaenoic acid (DHA) as an adjuvant therapy in the treatment of breast cancer patients slowed tumor growth, increased patient survival, and reduced treatment-related side effects [93]. Therefore, it is necessary to establish the most favorable ratio between the intake of n-3 and n-6 PUFAs, particularly due to their role in eicosanoid synthesis and their impact on cancer development and treatment [94,95].

Among dietary regimens, the ketogenic diet, a low-carbohydrate and high-fat diet, seems to possess the ability to attenuate tumor growth. It was reported that in experimental animal studies, a low-carbohydrate ketogenic diet increases the efficiency of phosphoinositide 3-kinase (PI3K) inhibitors and synergistically reduces the growth of PIK3CA-mutant tumors [96]. Under ketogenic conditions, encapsulated gemcitabine yielded notable pro-apoptotic effects in MIA-PaCa-2 cells, showing a synergistic effect that can hold promise for improving therapeutic options for pancreatic cancer [97]. Using in vitro cell lines and in vivo xenograft metastasis mouse models, it was recognized that a ketogenic diet enhances the response of estrogen receptor-positive liver metastatic breast cancer to fulvestrant treatment [98]. Compared with normal cells, breast cancer liver metastatic cells had more active pathways associated with glucose metabolism, and in affected animals, enhanced glycogen hepatocyte deposition was observed. The data available in the scientific literature on preclinical models and data derived from clinical observations suggest predominantly favorable effects of the ketogenic diet as an integral part of cancer treatment, but with significant variability in outcomes due to important methodological differences, including the type of tumor, timing of intervention, and diet composition. Moreover, clinical observations are based on relatively small groups of patients, which makes it difficult to conclude. Currently, many clinical trials are being conducted to assess the effectiveness of the ketogenic diet in cancer treatment, including the following:

- Phase II trial in patients with metastatic pancreatic cancer receiving chemotherapy (NCT04631445),
- A ketogenic diet pilot study for overweight prostate cancer patients on active surveillance (NCT03194516),
- A pilot presurgical trial of insulin inhibition by a ketogenic diet in operable breast cancer to assess the effect of a low-fat diet with extra fiber, fruits, and vegetables, and a ketogenic diet low in carbohydrates, on breast tissue in women with ER^+^ or ER^−^ breast cancer (NCT02744079),
- Single-center trial on ketogenic diet and immunotherapy in advanced cancer; this study evaluates the safety and effects of a ketogenic diet combined with immunotherapy in adults with advanced melanoma, cutaneous squamous cell carcinoma, or renal cell carcinoma (NCT06896552),
- A ketogenic diet therapy in patients with acromegaly (NCT06949891),
- Effectiveness of a ketogenic diet in MELAS (mitochondrial encephalomyopathy, lactic acidosis, and stroke-like episodes) syndrome (NCT06013397),
- Impact of a ketogenic diet intervention during radiotherapy on body composition (NCT02516501),
- A phase II study on the ketogenic diet vs. standard anticancer diet guidance for patients with glioblastoma in combination with standard-of-care treatment (NCT05708352).

It should be noted that our analysis of studies included in the clinical trial website (clinicaltrials.gov) shows that many investigations into the ketogenic diet for cancer treatment have been terminated, withdrawn for unspecified reasons, or have an unknown status. This finding suggests that, although most published clinical observations indicate the potentially beneficial effects of various types of ketogenic diets for patients with cancer, the use of specific ketogenic diet versions in clinical practice may be associated with adverse effects and may be burdensome for patients. Therefore, properly designed and controlled clinical trials are necessary to assess the potential of using specific variants of ketogenic diets in the treatment of various cancers.

Available clinical data indicate that the ketogenic diet can support the current standard therapeutic approaches for glioblastoma treatment and increase their efficacy. Recently, the evidence-driven framework for observational and interventional clinical studies on ketogenic metabolic therapy in glioblastoma has been proposed [99]. The properly designed ketogenic therapy can normalize the tumor environment and reduce the proliferation of glioblastoma cells by increasing substrate competition and targeting glycolysis and glutaminolysis. Furthermore, the proposed ketogenic diet approach may also serve as an effective adjuvant therapy in other tumors that are similarly dependent on glycolysis and glutaminolysis [100,101]. However, the efficacy of this therapeutic approach awaits confirmation in well-controlled interventional clinical trials.

The ketogenic diet leads to the production of ketone bodies, bioactive molecules synthesized in the liver and used peripherally as an alternative energy source when glucose availability is low. Current research primarily focuses on the ketone body metabolism within tumors and their roles in molecular signaling, inflammation, and oxidative stress [102]. Nevertheless, little is known about the influence of individual ketone bodies on the metabolism and development of different types of cancer, gene expression, epigenetic modifications, and interactions with various cancer therapies used in clinical practice and their efficacy. Both short- and long-term outcomes of a well-characterized ketogenic diet in patients with various cancers need to be systematically evaluated. It is important to note that a ketogenic diet may be associated with side effects and health risks. These may include micronutrient deficiencies; cardiovascular disease; hepatic, renal, and pancreatic complications; as well as fatigue, headaches, nausea, weight loss, osteoporosis, and cognitive or mood disturbances such as confusion and irritability [103,104,105]. Moreover, these side effects may synergize with those of chemotherapy, raising additional concerns.

## 4. Amino Acid Metabolism in Tumors

Glucose is accumulated as glycogen and fatty acids as triacylglycerols, but a.a.s. are not stored in the body. While many proteins are synthesized for their functional roles rather than for storing excess a.a.s., their metabolism plays a crucial role in tumor growth. In 1955, Eagle et al. [106] found that tumor cell survival was reduced under Gln deficiency, marking the beginning of attention to a.a.s. metabolic reprogramming in cancer. Fifty-six years later, Hanahan and Weinberg confirmed that metabolic reprogramming, alongside immune escape, is a hallmark of tumors [2,107].

In mammalian cells, a.a.s. are carbon and nitrogen donors in the biosynthesis of the majority of compounds, so they can influence tumor growth and progression. The enhanced a.a.s. anabolism is characterized for tumor growth, while the metastatic tumor cells, to survive stress, need energy generated mainly by catabolic pathways. The metabolism of 20 standard proteinogenic a.a.s. has been extensively studied to control specific tumor growth. There is growing recognition that significantly altered a.a. levels in the tumor microenvironment, along with the increased demand for specific a.a.s. by tumor cells, play an important role in cancer development. Tumor cells often aggressively compete for and consume nutrients available in the microenvironment. As a result, there is a shortage of nutrients for the tumor stromal cells. These alterations can facilitate the aggressive growth and multiplication of tumors [108]. Consequently, it has been proposed that inhibiting a.a. transporters to restrict a.a. uptake (Table 1) may represent a promising path to therapeutic success [109,110,111]. However, it is important to remember that a.a. metabolism may be altered differently in various cancers. Therefore, research is needed in this area to understand their role in cancer treatment.

### 4.1. Glutamine

In cancer, Gln serves as a source for at least 50% of nonessential a.a.s. used in protein synthesis [112] and exhibits a distinct metabolism pattern in cancer patients. Gln levels can increase during the later stages of malignancies such as prostate cancer, leukemia, and ovarian cancer, whereas early-stage colorectal and breast cancers often exhibit a tendency toward Gln deficiency [113,114,115,116,117]. To sustain cell growth and proliferation, tumors alter both the consumption and processing of Gln, with many types of neoplasms exhibiting Gln addiction. The first reports on the high Gln demand in cancer were published in 1955. The author observed that human carcinoma cells required twice as much Gln as normal L-strain fibroblasts and that the Gln requirement in HeLa cells was 10 to 100 times greater than that of any other a.a. [106]. Then, Reitzer at al. confirmed the role of Gln, indicating that Hela cells mainly consume Gln for nearly all of their energy production, even in the presence of high glucose levels [118]. Similarly, cancer cells from hepatomas and fibrosarcomas showed a 5–10-fold higher rate of Gln consumption than normal hepatocytes [119]. This is particularly evident in most of the immortalized cell lines widely used in research, which exhibit elevated Gln requirements. This intensified Gln utilization may contribute to the reduced serum Gln levels observed in colorectal cancer patients, a condition associated with poorer prognosis [116,120,121,122]. Metabolomic analyses have shown that Gln is present at undetectably low levels in tumors when compared with healthy tissue [123], and cancers exhibit varying Gln concentrations across different intratumoral areas, with Gln levels being particularly low in the central region of glioblastoma multiforme compared with the outer regions [124,125]. This may suggest an inability of Gln to reach the poorly vascularized core. Cancer cells that are deprived of Gln usually arrest in S-phase [126]. In pancreatic and breast cancers, Gln, after glucose, is a rapidly utilized nutrient. Gln can be used by cancer cells mainly to supply carbon and nitrogen sources for the synthesis of nucleic acids and glutathione as well as acting as a substrate for glucose and a carbon donor for the synthesis of lipids [127].

**Figure 3:**
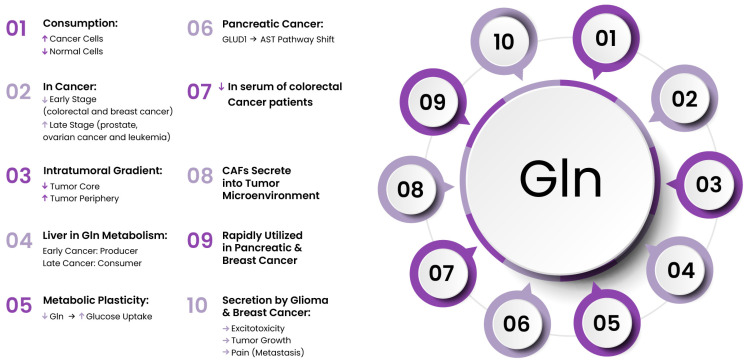
Stage- and tissue-specific patterns of glutamine metabolism in cancers.

In canonical Gln metabolism, the Krebs cycle is supported by Gln that turns into glutamate (Glu) and then undergoes oxidative deamination to form α-KG via glutamate dehydrogenase (GLUD1). α-ketoglutarate then feeds into the TCA cycle. The malate–aspartate shuttle functions alongside the TCA cycle to transport reducing equivalents from glycolysis into the mitochondria, supporting oxidative phosphorylation. However, in pancreatic cancer, Gln metabolism is reprogrammed and differs from the traditional α-KG production in the mitochondrial pathway, shifting instead toward processing through the aspartate aminotransferase pathway. A characteristic feature of this reprogramming is the increased expression of aspartate aminotransferase (AST, also known as GOT) and the suppression of GLUD1, driven by mutant Kras. The repression of GLUD1 promotes AST-mediated generation of aspartate (Asp) in the mitochondria. Then, Asp is released into the cytosol and converted to pyruvate with NADPH production [128]. Additionally, in cancer, the altered Gln metabolism in the liver is complicated and depends on progressive malignant growth. Initially, the liver acts as a primary producer of Gln, releasing it into the bloodstream through a Gln transporter system independent of Na^+^. However, in advanced-stage cancer, there is modulation of hepatic a.a. transport through the induction of membrane expression of System N, an influx carrier, which makes the liver a Gln consumer (Figure 3) [129,130,131].

Beyond its role in energy metabolism, the α-nitrogen of Gln also serves as a donor in transamination reactions, contributing to the biosynthesis of several a.a.s., including alanine, aspartate, serine, proline, and ornithine, or of polyamines [132,133,134,135,136]. Gln is involved in the uptake of essential a.a.s. such as leucine from the extracellular environment through the antiporter SLC7A5, which exports glutamine out of the cell [137]. It has proven to play a crucial role in cancer cell growth, as both pharmacological inhibition of SLC7A5 or its genetic silencing lead to a reduction in the proliferation of cancer cells and tumor growth in xenograft models [138]. Additionally, another Gln transporter, SLC1A5, is highly expressed in triple-negative breast cancer patients, correlating with poor survival in tumor-bearing mice. It was demonstrated that blocking SLC1A5 greatly hindered tumor growth and migration in a subcutaneous xenograft model with renal carcinoma cells [139].

α-ketoglutarate, derived from Gln, is considered a cofactor for histone demethylases. Low Gln levels, similarly to glucose restriction, inhibit the activity of JmjC-KDMs (Jumonji C domain-containing histone lysine demethylases), resulting in excessive histone hypermethylation in the tumor core. The changes in histone methylation and gene expression accompanying Gln depletion contribute to the development of drug resistance in melanoma cells to the BRAF inhibitor [124]. It can be hypothesized that there is an interaction between Gln and glucose metabolism in actively proliferating cells. The administration of V-9302, an inhibitor of SLC1A5 that reduces Gln availability, increases glucose uptake in cancer and immune cells in allograft models [140]. This report highlights how plastic the tumor mass is; a deficiency of one component leads to the rerouting of metabolism to utilize other available compounds. Selective allocation of these nutrients to specific cell types could be used to develop therapies.

Additionally, Glu, derived from Gln, can be converted to glutathione by the enzymes glutamate–cysteine ligase and glutathione synthetase. Therefore, Gln and its derived metabolites can regulate redox balance, gene transcription, and intracellular signaling [141,142]. Thus, Gln deprivation resulted in a significant increase in oxidative stress in pancreatic cancer [128]. The pool of intracellular Gln is involved in the activation of mTORC1 signaling, which is a regulator of cell growth, as well as the prevention of apoptosis [143]. On the other hand, it has been reported that in mice, Gln utilization by lung tumors and healthy lung tissue was similarly low [144].

Glutamine secretion from glioma cells can contribute to excitotoxic brain injury and tumor expansion. The potential of phenylglycine derivatives to selectively block Glu release from glioma cells presents promising clinical opportunities [145]. The ongoing clinical trial assesses the efficacy of anti-glutamatergic treatment with gabapentin, sulfasalazine, and memantine, in combination with standard chemoradiotherapy, in patients with newly diagnosed glioblastoma (NCT05664464). Additionally, pain can arise from disrupted glutamatergic signaling. Breast cancer cells, similarly to glioma cells, release elevated levels of Glu and, additionally, frequently metastasize to bone. Exogenous Glu may interfere with normal bone remodeling and contribute to cancer-related bone pain, as shown in a preclinical study. The use of the pharmacological inhibitor sulfasalazine, which blocks Glu release, may have a beneficial effect [146]. Interestingly, there is increasing evidence about the involvement of Glu signaling in human malignancy [147]. Intense research is being conducted regarding the role of CAFs in the secretion of high levels of Gln into the tumor microenvironment. All the aforementioned features make Gln an attractive target in anticancer therapy.

In the 1990s, acivicin, DON, and azaserine, analogs of L-glutamine, were tested as mimetic antimetabolites [148]. These compounds interfere with the transfer of nitrogen or sulfur and disrupt nucleic acid biosynthesis. However, their clinical application ultimately failed due to gastrointestinal and neurotoxicity, with myelosuppression also being observed. In contrast, DRP-104, a DON peptide prodrug, is currently undergoing clinical trials and has been granted FDA Fast Track designation for the treatment of advanced non-small-cell lung cancer with KEAP1 (Kelch-like ECH-associated protein 1), NFE2L2 (Nuclear Factor, Erythroid 2 Like 2), and/or STK11 (Serine/Threonine Kinase 11) mutations in patients who have received prior therapy (NCT04471415). DRP-104 reduces glutamine-dependent nucleotide synthesis and, as a result, suppresses KEAP1 mutant tumors [149].

In cancer, many genes that encode glutamine-related enzymes of metabolic chains are mutated, which makes cancer more Gln-dependent than normal tissue. In cancers, the expression of enzymes connected with Gln metabolism is different and dependent on the cancer tissue origin and oncogenotype [150]. Additionally, the isozymes glutaminase 1 (GLS1) and GLS2 have opposing roles in tumors: GLS1 exhibits oncogenic properties, whereas GLS2 functions as a tumor suppressor [151,152]. The elevated expression of GLS1 has been shown in multiple cancer types and has generated significant interest over the past decade. However, the function of GLS1 in cancer has not been fully explained, but it is clear that its inhibition blocks Gln metabolism. Gln cannot be transformed into glutamate and α-KG, inhibiting the TCA cycle and energy production. A few selective GLS1 inhibitors have been developed for the treatment of patients with glutamine-dependent cancers. CB839 (Telaglenastat) has shown antitumor activity in triple-negative breast cancer and hematological malignancies in preclinical studies [153] and is currently in multiple clinical trials, including in patients with solid tumors; renal carcinoma; and colorectal, lung, and cervical cancers (NCT03875313, NCT04250545, NCT03965845, NCT03428217, NCT05521997). Another GLS1 inhibitor, IPN60090 (IACS-6274), was tested in patients with advanced solid tumors (NCT05039801) [154], and a blocker of “kidney-type” glutaminase, bis-2-(5-phenylacetamido-1,2,4-thiadiazol-2-yl)ethyl sulfide (BPTES), inhibited the cancer growth in P493 tumor xenografts [155,156]. Moreover, UPGL00004 is a new agent with a similar binding affinity to glutaminase C as CB-839, and it more potently inhibits the activity of glutaminase C than BPTES. UPGL00004 effectively inhibits the proliferation of triple-negative breast cancer cells as well as reducing tumor growth when combined with bevacizumab [157]. On the other hand, clinical trials are investigating the use of intravenous or oral Gln intake to reduce chemotherapy-induced toxicity in breast cancer (NCT00772824). Similarly, several studies have demonstrated that administering intravenous alanyl–glutamine to colon cancer patients can alleviate chemotherapy-related side effects [158,159].

### 4.2. Serine and Glycine

Extracellular Ser is second only to Gln among a.a.s. consumed by cancer cells, as revealed by metabolite profiling of 60 different cancer cell lines [160]. Serine serves as a precursor for proteins, nucleic acids, and phospholipids as well as supporting tumor antioxidant defense. Through serine hydroxymethyltransferase (SHMT), Ser is converted to glycine (Gly) and one-carbon units for carbon metabolism.

Normal cells typically have a lower demand for Ser and Gly, but many cancers (such as melanoma, breast, and lung cancer) become dependent on their de novo production, both intrinsically and in response to cancer treatments [161,162]. In addition to de novo Ser/Gly synthesis, these metabolites can also be obtained from the microenvironment. The Ser/Gly synthesis pathway is overexpressed in about 30% of all cancers [163]. In neuroendocrine prostate cancer, MYCN-amplified neuroblastoma, colorectal cancer, and breast cancer, many metabolic enzymes of the biosynthesis of Ser and Gly are frequently upregulated; thus, innovative therapy targets Ser and Gly biosynthesis [164,165,166]. In addition to MYC, the tumor suppressor p53, the oncogene KRAS, the mTOR-ATF4 axis, and the T-cell leukemia-associated R98S mutation have also been shown to promote Ser/Gly synthesis in cancer cells [167,168,169,170]. Gly uptake and the expression of mitochondrial enzymes in Gly biosynthesis correlate with the proliferation of NCI-60 cells [171]. Breast cancer relies on Ser/Gly synthesis, with 6% of patient samples showing copy number gains of the phosphoglycerate dehydrogenase gene (*PHGDH*). PHGDH is responsible for catalyzing the first step in Ser synthesis. Moreover, 70% of estrogen receptor-negative breast tumors exhibit elevated PHGDH protein levels, and its inhibition reduces cancer cell survival [172,173]. However, the clinical use of PHGDH inhibitors is restricted because Ser plays a vital role in central nervous system development, and a deficiency in PHGDH can result in neurological defects [174]. The expression levels of serine hydroxymethyltransferase 2 (*SHMT2*) are positively associated with the grade of breast cancer [175]. Moreover, increased Ser consumption enhances DNA damage repair in 5-FU-resistant colorectal cancer cell lines [176].

Preclinical studies have shown that blocking both exogenous and endogenous sources of Ser in malignant cells is associated with superior anticancer effects compared with limiting them alone [177]. Inhibition of tumor cell proliferation through the restriction of Ser and Gly intake has been observed in intestinal cancer and lymphoma in mice [169]. Recent reports indicate that sertraline, a widely used antidepressant in psychiatry, reduces SHMT1/2 activity and could potentially serve as an agent targeting cancers dependent on Ser/Gly synthesis [178]. Last year’s report highlights that sertraline increases radiosensitivity and boosts the effectiveness of treatment in a non-small-cell lung cancer lines [178].

The promising effects of sertraline are currently being confirmed in several ongoing clinical studies. Among them is a trial exploring the safety of a combined treatment approach using sertraline and metronomic temozolomide in recurrent glioblastoma (NCT02770378) as well as a trial investigating the combination of tamoxifen and sertraline in women with breast cancer or at high risk of developing it (NCT00667121). There is also a study where sertraline is given with timed-sequential cytosine arabinoside in patients suffering from relapsed and refractory acute myeloid leukemia (NCT02891278).

### 4.3. Branched-Chain Amino Acids

BCAAs are exogenous a.a.s.—leucine, isoleucine, and valine—with implications in tumorigenesis. Although they have been associated with the formation of various types of tumors, the effect of dietary BCAA consumption on the spread of cancer is not yet fully understood. Cancers tend to preferentially absorb BCAAs; for instance, pancreatic ductal adenocarcinoma cells consume 1.5 to 2.5 times more BCAAs than immortalized normal cells [179]. Additionally, the increased plasma levels of leucine, isoleucine, and valine are connected to a risk of pancreatic cancer in the future that is more than twice as high and are also characteristic of early-stage pancreatic cancers driven by mutant Kras expression [180]. An elevation in BCAA levels has also been observed in plasma and tissue in breast cancer [181].

The balance of BCAAs is regulated by their catabolic process. Overexpression of the enzymes responsible for the first step in BCAA degradation has been observed in several tumors, including pancreatic cancer and glioma [182,183]. BCAAs are transaminated into branched-chain ketoacids by specific aminotransferases (BCAT1 and BCAT2) and subsequently catabolized by alpha-ketoacid dehydrogenase (BCKDH) to produce intermediates for the TCA cycle, generating NADH in the process. Branched-chain alpha-ketoacid dehydrogenase kinase (BCKDK) inhibits BCKDH through phosphorylation at the Ser 293 residue. Glutamine, which is synthesized from BCAA catabolism, is a vital a.a. required by numerous tumor cells, as described above. To maintain appropriate levels of Gln, cancer cells enhance the expression of *BCAT1* [184]. The potential mechanism of promoting cell growth by BCAA is the activation of the mTORC1 pathway [185]. In breast cancer, BCAT1 can trigger the mTORC1 pathway, leading to increased mitochondrial biogenesis and ATP synthesis, which provide energy for growth and colony formation of tumor cells [181].

In cancer, the metabolic reprogramming of BCAAs is determined by changes in the expression as well as the activity of BCAA transporters and the enzymes that are involved in their catabolism [186]. Thus, BCAT1, BCAT2, and BCKDK have emerged as potential therapeutic targets for tumor treatment. However, different phenotypes of malignancy are associated with various isoforms of enzymes [187]. It has been reported that BCAT is regulated by oncogenes in malignancy [188]. Moreover, *BCAT1* was overexpressed in malignant melanoma patients and mouse malignant melanoma cells. The inhibition of the proliferation and migration of melanoma cells was observed in *BCAT1* knockdown [189]. Overexpression of *BCAT1* was also observed in gliomas [183], ovarian [190], liver [191], bone sarcomas [192], and endometrial cancer [193]. Moreover, the levels of BCAT1/2 are upregulated, while BCKDH is decreased, in hepatocellular carcinoma [191]. However, not every type of cancer is characterized by overexpression of *BCAT1,* and suppression of *BCAT1* inhibits proliferation in not all types of tumor [194]. In humans, BCAAs cannot be synthesized; thus, the transporters (SLC7A5, SLC7A8) responsible for their uptake are essential, and they are highly expressed in glioblastoma and renal cell carcinoma. The drug JPH203, which targets this transporter, has already been tested in a preclinical study involving prostate cancer resistant to cabazitaxel [195].

The latest research found no link between higher dietary BCAA intake and an increased risk of colorectal cancer [196]. However, a separate study on colorectal cancer patients revealed a positive association between high BCAA intake and an elevated risk of mortality [197].

Changes in BCAA levels in plasma and tissue are accompanied by an elevated expression of *BCAT1* in breast cancer. The knockdown of *BCAT1* resulted in reduced growth and colony formation in MCF-7 cells [181]. In turn, it has been shown that in mice on high-BCAA diets, the growth of breast cancer and lung metastases is suppressed. These effects are linked to a reduction in N-cadherin expression [185]. Considering the widespread availability of commercial BCAA products, these findings suggest that increasing BCAA intake could offer a practical dietary strategy to slow tumor progression and enhance breast cancer treatment [185].

However, caution should be exercised due to the dual role of BCAAs in tumors, which presents significant challenges and limitations. Unexpectedly, it has been shown that a deficiency in BCAAs can promote cancer progression [198]. Oral BCAA supplementation, as the simplest method to influence BCAA metabolism in vivo, has been investigated over the past decade for its potential to reduce fibrosis, inhibit tumor growth, and enhance survival rates in animal models of liver cirrhosis [199,200]. On the other hand, a diet rich in BCAAs stimulates the progression of pancreatic intraepithelial neoplasia in *LSL-KrasG12D/^+^*; *Pdx1-Cre* (KC) mice [201]. However, given the inconsistencies in this area, inhibiting BCAA enzymes may not be effective in all cancers, while in others, it could be beneficial. To obtain accurate results, further studies are needed, ideally with larger cohorts.

Many clinical trials (NCT03908255, NCT06604910, NCT01434524) have explored the effects of BCAA supplementation on disease progression, and many of them are still ongoing. It has been proven that BCAAs lead to an increase in event-free survival, reduce the complications in hepatocellular carcinoma patients [202,203], and provide positive outcomes for patients with advanced liver cirrhosis [204]. Another study (NCT01434524) is examining the role of long-term oral BCAA supplementation in preventing liver tumorigenesis in patients undergoing liver resection. Meanwhile, the latest studies from 2024 assess whether oral BCAA supplementation can help minimize muscle loss, impairments in swallowing function, and short-term complications in patients with bone cancer (NCT06604910).

### 4.4. Arginine

Arginine (Arg) is a semi-essential a.a. that is associated with cell proliferation and signaling. It is derived from internal sources like the urea cycle and from external sources such as the diet. However, due to the silencing of argininosuccinate synthase 1 (ASS1), certain tumors lose the ability to synthesize Arg, presenting a promising opportunity to combat cancer. ASS1 downregulation has been associated with advanced tumor stages [205]. In contrast to normal cells, melanoma, hepatocellular carcinoma, prostate, and pancreatic cancers are dependent on external Arg, which regulates mitochondrial activities in tumor metabolism. Arginine acts as a fundamental component of proteins and is a key precursor in the production of nitric oxide (NO), ornithine, agmatine, glutamine, and creatine [206,207].

Recently, it has been documented that colorectal cell lines cannot grow in medium without Arg supplementation [208]. In both primary and metastatic colorectal tumors, an increase in the synthesis of arginine transporters has been observed. This suggests that cancer cells have a heightened demand for this a.a. [209,210]. Moreover, the knockdown of the arginine transporter can decrease breast cancer cells’ viability as well as induce their apoptosis [211].

Arginine can be hydrolyzed by arginases (ARGs). In mammals, these enzymes exist in the form of cytoplasmic ARG1 and mitochondrial ARG2 isozymes. In cancers such as glioblastoma, thyroid tumors, prostate cancer, and breast cancer, both isozymes are upregulated to produce polyamines, which are responsible for proliferation and DNA stability [212,213,214,215,216]. In mice with early-stage breast cancer metastasis, a shift in L-arginine metabolism was observed, favoring the hydrolysis of L-arginine to L-ornithine and increased polyamine synthesis. Late-stage metastasis is characterized by an additional increase in the plasma concentration of asymmetric dimethylarginine [217]. In skin, colon, breast, hematologic, and prostate malignancies, high ARG activity has been documented [218,219,220]. In patients with primary colorectal cancer, ARG activity increases threefold, and in those with metastatic colorectal cancer, it increases sixfold. This rise in ARG activity is accompanied by a twofold increase in serum L-arginine concentration. Interestingly, in non-cancer conditions associated with increased ARG activity, serum L-arginine concentrations are typically reduced [221]. Additionally, Arg can shift T-cell metabolism from glycolysis to oxidative phosphorylation, promoting their antitumor activity [222]. Therefore, the use of ARG inhibitors to counteract immune suppression seems reasonable.

Interestingly, various studies have shown that the migration of cancer cells is impaired by Arg deprivation. A plausible explanation lies in the fact that nitric oxide (NO) and polyamines, key products of Arg metabolism, play a significant role in promoting and regulating cellular motility. However, their precise roles are still not fully understood. In ovarian and pancreatic cancer cell lines, cell cycle arrest at the G0/G1 phase was observed following treatment with pegylated recombinant human Arginase I cobalt [HuArgI (Co)-PEG5000]c [223]. Moreover, depending on the tumor type, there are various mechanisms of cell death, ranging from apoptosis and necroptosis to autophagy, when deprived of Arg [223,224,225]. In the case of colorectal cancer cell lines, Arg depletion decreased cancer cell motility, invasion, and cell adhesion, but these effects were rescued by the addition of L-citrulline, a precursor of arginine [226]. The anticancer properties of pegylated arginine deiminase (ADI-PEG20) and pegylated ARG have been observed in a variety of cancers [227,228,229]. Currently ADI-PEG20 has undergone several clinical trials in patients with hepatocellular carcinoma, mesothelioma, and metastatic melanoma, showing promising outcomes. Despite promising results in phase I/II trials, the phase III trial of ADI-PEG20 in 635 hepatocellular carcinoma patients failed [230]. Additionally, the trial of ADI-PEG20 in patients with relapsed sensitive or refractory small-cell lung cancer was discontinued early due to insufficient efficacy and slow patient enrollment (NCT01266018).

In turn, clinical trials of pegylated ARG in cancer patients are not so numerous. At present, recombinant human arginase (PEG-BCT-100) is being evaluated for its safety and efficacy in treating advanced hepatocellular carcinoma (NCT01092091; NCT00988195) as well as in solid tumors including melanoma, renal cell carcinoma, prostate cancer, and hepatocellular carcinoma (NCT02285101) and in brain cancers (NCT03455140).

### 4.5. Cystine and Cysteine

Cysteine (Cys) is classified as a nonessential amino acid, yet it plays a crucial role in cellular metabolism, particularly in carbon and sulfur transformation. Its significance becomes more apparent during periods of heightened nutritional need, and tissues primarily acquire it in the form of cystine, the oxidized version of Cys. However, leukemia and breast cancer cells can preferentially uptake Cys directly [231,232]. Colorectal tumors are also rich in Cys compared with adjacent non-tumor tissue, supporting the hypothesis that cancer cells exhibit a dependency—or “addiction”—to cysteine [233].

In addition to being a constituent of the tripeptide glutathione, as well as a precursor to proteins and taurine, Cys also serves as a source of energy and biomass. In contrast to non-transformed tissues, many cancers rely on external cystine for proliferation and to maintain cell viability. Cysteine allows ovarian cancer cells to survive in hypoxic environments and evade carboplatin-induced cytotoxicity [234].

There was observed a positive correlation between the Cys level in plasma and breast cancer risk [235]. A recent study documented that N-acetylcysteine supplementation accelerates tumor progression and reduces survival in transgenic lung cancer in mice [236]. It was proved that in the nucleus pulposus, homocysteine promotes oxidative stress and ferroptosis mediated by the upregulated methylation of glutathione peroxidase 4 (*GPX4*) [237]. Additionally, Cys deficiency inhibits ovarian clear cell carcinoma in both in vitro and in vivo models [238]. Consequently, the regulation of cysteine intake through diet has gained significant attention in recent years.

SLC7A11/xCT, a major transporter of Cys, is reported to be highly expressed in many tumor cells and correlates with poor survival in prostate, breast, and thyroid cancer patients. Intensified Cys transport allows cancer cells to develop chemoresistance [239,240]. Contrary to solid tumors, the xCT transporter is downregulated in chronic lymphocytic leukemia [241].

Erastin is the first identified selective inhibitor of Cys transporters that induces ferroptosis. Even a brief exposure of human ovarian cancer cells to erastin increased the cytotoxic effects of cisplatin [242]. Additionally, inhibition of xCT has been shown to increase the sensitivity of HeLa cells to chemotherapies [243]. Another study revealed that inhibiting cystine or Cys transporters impedes the growth of both colorectal cancer cells and tumors [233]. Moreover, it has been documented that both Cys and *SLC7A11* are critical for pancreatic ductal adenocarcinoma growth, and Cys depletion induces the death of pancreatic tumor cells in mice [244]. Studies have indicated that enhanced activity of the endogenous transsulfuration pathway may help cancer cells survive in conditions with low extracellular Cys levels. Despite its preclinical promise, xCT inhibition proved ineffective in a clinical trial for glioma treatment. Sulfasalazine, an inhibitor of the xCT-Cys/Gln antiporter, had a detrimental impact on patient survival, leading to the early termination of the trial [4]. Currently, new clinical trials are recruiting participants to assess the potential efficacy and safety of sulfasalazine in patients with metastatic colorectal cancer (NCT06134388) and acute myeloid leukemia (NCT05580861).

**Table 1:** Summary of various amino acid-targeted metabolic therapeutic approaches in the fight against cancer.

Interventions Targeting Amino Acid Metabolism	
Amino Acids	Effects
**Glutamine**	-Alanyl-glutamine dipeptide reduces chemotherapy-related side effects -Glutamine antagonist suppress KEAP1-mutant tumors -Inhibition or genetic silencing of glutamine transporters (SLC7A5, SLC1A5) reduces tumor growth and migration -Glutaminase 1 inhibitors treat glutamine-dependent cancers
**Serine and Glycine**	-Inhibition of PHGDH reduces cancer cell survival -Increased serine consumption enhances DNA damage repair in colorectal cancer cells -Blocking sources of serine is associated with anticancer effects -Restriction of serine and glycine intake inhibits the proliferation of intestinal cancer and lymphoma -Sertraline: reduces SHMT1/2 activity, increases radiosensitivity, and enhances treatment effectiveness in non-small cell lung cancer
**Branched-Chain Amino Acids**	-BCAT1 knockdown inhibits melanoma cell proliferation and reduces growth and colony formation in breast cancer -SLC7A5 inhibitor suppresses growth of prostate cancer cells -High-BCAA diets reduce breast cancer growth and lung metastasis, showing positive outcomes in advanced liver cirrhosis -Diet rich in BCAA stimulates the progression of pancreatic intraepithelial neoplasia -BCAA supplementation increases event-free survival and reduces complications in hepatocellular carcinoma
**Arginine**	-Knockdown of arginine transporter reduces breast cancer cell viability -Pegylated recombinant human arginase I cobalt (HuArgI (Co)-PEG5000) induces cell cycle arrest at the G0/G1 phase -Arginine depletion in colorectal cancers decreases cell motility, invasion, and adhesion -Pegylated arginine deiminase (ADI-PEG20) and pegylated arginase exhibit anticancer properties.
**Cysteine**	-Cysteine enables cancer cells to survive in hypoxic conditions -N-acetylcysteine supplementation accelerates tumor progression and reduces survival in transgenic lung cancer models -Cysteine deficiency inhibits ovarian clear cell carcinoma -Inhibition of cysteine transporter induces ferroptosis and boosts cisplatin′s cytotoxic effects in ovarian cancer cells -Blocking xCT increases sensitivity of HeLa cells to chemotherapies and impedes the growth of colorectal cancer -Cysteine depletion induces death in pancreatic tumor cells

## 5. Clinical Translation of Metabolic-Targeted Agents

Bridging the gap between laboratory findings and patient care, several metabolism-targeted agents have progressed into clinical development and offer new hope in oncology. The TCA cycle, often mentioned above, is a fundamental metabolic pathway comprising a series of enzymatic reactions that facilitate the oxidation of sugars, amino acids, lipids, and other substrates to generate the cellular energy necessary to survival. The isocitrate dehydrogenase (IDH) enzyme family serves as a rate-limiting catalyst, playing a key role in converting isocitrate to α-KG. Notably, the isoforms IDH1 and IDH2 have been linked to tumor development. Extensive research efforts targeting mutant IDH1 culminated in the FDA’s 2022 approval of ivosidenib (synonym: AG-120; brand name: Tibsovo) for a supplemental indication, expanding its use in combination with azacitidine for patients with newly diagnosed IDH1-mutated acute myeloid leukemia (supported by phase III clinical trials such as NCT03173248 and the phase Ib/II trial NCT02677922). It is important to note that studies have demonstrated an association between IDH1 mutations and the initiation and progression of several other malignancies, including glioblastoma and chondrosarcoma. Currently, there are many other potential inhibitors of mutant IDH1; however, most of these small molecules still face challenges such as insufficient in vitro activity and poor drug gability. The clinical indications for ivosidenib continue to expand, with recent data demonstrating that in October 2023, the FDA approved it for the treatment of adult patients with relapsed or refractory myelodysplastic syndromes (MDSs) harboring a susceptible IDH1 mutation [245,246]. Additionally, ivosidenib’s approval for IDH1-mutated cholangiocarcinoma was supported by the completed phase III ClarIDHy trial (NCT02989857).

Upregulated Gln intake to drive the TCA cycle is one of the strategies employed by cancer cells to support their rapid growth and survival. The discovery that renal carcinoma cells (RCCs) are highly dependent on Gln formed the basis for studies investigating the use of a glutaminase inhibitor, CB-839. Despite the unfavorable outcome of the CANTATA trial in RCC, the cabozantinib combination may still offer advantages for select populations. In May 2018, the FDA granted Fast Track designation to CB-839 in combination with cabozantinib for the treatment of metastatic RCC in patients who had previously received one or two lines of therapy. However, the current phase I/II trial evaluating the safety and efficacy of CB-839 in combination with nivolumab in patients with advanced solid tumors has not demonstrated therapeutic efficacy according to the latest findings reported this year [247,248].

Etomoxir, a CPT1 inhibitor, represents a potential therapeutic option for patients with malignant glioma. By disrupting the FAO pathway, it has shown promising results in preclinical models, including reduced tumor growth and improved treatment outcomes. Etomoxir was previously investigated in phase II trials for heart failure and type 2 diabetes; however, the studies were discontinued due to adverse effects, particularly elevated liver transaminase levels. In 2025, Numiera Therapeutics, a United States-based pharmaceutical company, announced that etomoxir had been granted orphan drug status for the treatment of malignant glioma, and clinical trials are already planned [249].

Agents such as ivosidenib, CB-839, and etomoxir (Table 2) represent key milestones in the translational journey from metabolic research to clinical application, each targeting distinct metabolic vulnerabilities within tumors. These achievements demonstrate that the direction of research and investigations in this field is increasingly on the right track.

## 6. Conclusions and Future Perspectives

The development of cancer requires the modulation of key cellular metabolic processes, enabling malignant cells to adapt to environmental conditions for their growth and survival. Metabolic reprogramming in tumor cells alters the utilization of glucose, fatty acids, and amino acids, affecting their requirement for specific nutrients. The TCA cycle highlights the metabolic role of certain a.a.s., such as Gln, in cancer progression. Glutamine contributes to ATP production and anabolic processes by entering the TCA cycle as α-ketoglutarate, thereby supporting the energy generation, redox balance, and biosynthesis required for tumor growth. This metabolic dependency becomes especially critical when cancer cells metastasize, requiring adaptation to differing resource availability between the primary tumor site and the new microenvironment. Cancer’s adaptability is driven by the high metabolic plasticity of malignant cells, which rapidly modulate their metabolism and simultaneously reshape their environment to suit their needs. For instance, Gln deprivation increases cancer cells’ reliance on alternative nutrients such as Asp, asparagine, BCAAs, or glucose. Conversely, prolonged inhibition of glycolysis may cause cancer cells to activate AMPK (AMP-activated protein kinase) signaling pathways, leading to upregulation of glutamine metabolism and/or increased fatty acid utilization (including β-oxidation, uptake, and storage), thereby promoting survival under glucose deprivation. In addition to that, in response to low oxygen levels, cancer cells induce the expression of glycolytic enzymes and *GLUT1*, which triggers autophagy and simultaneously helps them avoid apoptosis. These metabolic adaptations may explain some of the inconsistencies frequently observed in experimental findings. Metabolic phenotypes tend to vary according to the tissue of origin, tumor microenvironment, stage (primary versus metastatic tumors), and mutational differences. The metabolic flexibility of tumors, driven by real-time adjustments in metabolite levels, enzymatic fluxes, and the directionality of existing metabolic pathways, poses a significant obstacle to the development of effective cancer therapy. Understanding the role of various nutrients in tumor growth is a promising area of research that holds great potential for enhancing cancer treatment by strategically manipulating nutrient availability. The fight against cancer, therefore, requires the development of new therapeutic strategies that take into account and exploit the potential of the metabolic reprogramming of cancer cells. Achieving this goal demands a comprehensive understanding of the metabolic profiles across different cancer types.

Specific alterations, for example in a.a. metabolism, differ between tumor types and are shaped by numerous factors, including intrinsic cellular characteristics and components of the tumor microenvironment. This metabolic diversity underscores a major challenge in oncology: the heterogeneity of nutrient metabolism, such as a.a., throughout cancer development. Despite decades of research, our understanding of the mechanisms underlying this heterogeneity remains insufficient. The interactions between cancer cells and components of the tumor microenvironment further add to the context-specific complexity of a.a. and other nutrients’ metabolism. Within the tumor microenvironment, metabolic crosstalk between different cell types allows cancer cells to maintain continuous growth, even under challenging conditions. Notably, both cancer cells and immune cells rely on the same a.a.s., particularly Ser and Gln, and strategies that shift a.a. availability from cancer cells to immune cells could enhance antitumor immune responses. Cancer cells are also able to alter the nutrient makeup of the tumor microenvironment to promote their own growth while suppressing the activity of immune cells. Importantly, targeting the catabolic pathway of a specific a.a.s. can be beneficial for certain cancers, while targeting the same pathway in another cancer type may worsen prognosis. Some metabolic inhibitors can make cancer cells more sensitive to chemotherapy and radiotherapy by reducing the energy available, but they may also affect immune cell metabolism or cause side effects in healthy tissues.

The complexity of the metabolic networks in cancer underscores the need for new technologies to assess cancer’s metabolic heterogeneity. Future advancements may include liquid biopsy techniques to monitor circulating metabolites, exosomes, or other biomarkers, as well as the consideration of metabolic assays performed directly on patient tumor samples to guide precision or personalized therapy. Such heterogeneity spans various molecular levels, including the genome, epigenome, metabolome, lipidome, and proteome. It has significant clinical implications, including short- and long-term adverse outcomes and therapy resistance. Therefore, assessing these complex variations in both the tumor and its microenvironment is crucial for developing new, more effective therapeutic methods. This can be achieved by combining different clinical and biochemical diagnostic approaches, as has recently been proposed in the context of breast cancer [250]. The developed diagnostic strategy integrates in vitro and in vivo imaging, enabling the assessment of various aspects of tumor development and progression.

With up-to-date knowledge, several approaches are currently being developed to target a.a.s. metabolism, including their restriction, enzymatic depletion, and inhibition of specific metabolic pathways. However, it must be taken into account that strategies involving enzyme or transporter inhibition (Table 2) or dietary restriction of exogenous a.a.s. may also harm healthy tissues. Moreover, certain amino acid-derived metabolic intermediates can play dual roles, either promoting or inhibiting tumor growth, depending on the context. Numerous inhibitors of metabolic processes related to lipid and glucose utilization that are key to cancer have also been developed. However, when used alone, they are generally ineffective. Therefore, combined therapies that integrate traditional pharmacological agents or radiotherapy, metabolic inhibitors, and dietary regimens appear promising. Future clinical trials should evaluate the clinical effectiveness of such combined therapeutic approaches across various cancer types. The use of the ketogenic diet in cancer treatment as an adjuvant therapy is currently of great interest. However, the efficacy of this approach needs to be evaluated through ongoing and upcoming controlled clinical trials [100,251,252]. Supporting the patient’s overall health and minimizing the toxic and adverse effects of cancer therapy are of paramount importance. However, at least for now, no specific diet can be confidently prescribed to cancer patients due to a dearth of solid clinical evidence. A recent review and meta-analysis of published data examining the relationship between various diets (including a ketogenic diet; a Mediterranean diet; a plant-based, high-protein diet; and an anti-inflammatory diet) and chemotherapy toxicities and quality of life in patients undergoing treatment showed that there is insufficient evidence to determine which dietary intervention is most beneficial, and no intervention can be ruled out [253].

Another “piece of the puzzle” that cannot be overlooked when developing anticancer strategies supported by diet is the gut microbiota. Radiotherapy, chemotherapy, and immunotherapy all have the potential to alter a patient’s microbiome, while the composition of the microbiome itself can significantly impact how patients respond to these treatments. A large proportion of dietary intervention studies in cancer patients focus on the relationship between diet and the microbiome, aiming to understand how these interactions may influence clinical outcomes. However, many of these studies (NCT03341143, NCT03353402, NCT03637803, NCT04116775) are still enrolling participants or have yet to complete data analysis. Identifying diet–microbiota factors that influence solid tumor growth will enhance our understanding of cancer and support the development of more effective therapeutic strategies. In addition, variables such as ethnicity, environmental exposures, gender, and obesity may further influence the relationship between dietary strategies and treatment outcomes. Carefully evaluating these interconnected factors is essential to accurately determine how diet impacts therapy. Thus, significant challenges remain in translating microbiome research and the above-mentioned variables into clinical treatments. It seems once again that therapeutic strategies may need to be personalized, taking into account each patient’s unique microbiome profile and individual lifestyle characteristics. It offers hope for improved treatment efficacy and reduced toxicity through proper microbiota manipulation [254].

Future research is necessary to identify effective ways to support patients during and after treatment, with the goal of improving treatment efficacy and quality of life. In parallel, continued investigation using advanced technologies and precision medicine frameworks is needed to develop personalized treatment strategies. These strategies must be tailored to the type and stage of cancer, its microenvironment, and the metabolic profiles of individual patients. In future studies, cancer cell-type-specific metabolic profiling is essential to design an appropriate therapeutic strategy. A deeper understanding of the metabolic pathways critical for primary tumor growth and metastasis is also crucial. Ultimately, the question arises: how many metabolic hubs remain undiscovered? One promising approach for identifying relevant metabolic targets could involve unbiased CRISPR–Cas9 synthetic lethality screening of metabolic genes. Building on this concept, the integration of metabolomics and metabolic imaging into diagnostic workflows in cancer seems to be a promising direction. By combining metabolomic data with positron emission tomography (PET), magnetic resonance spectroscopy (MRS), or hyperpolarized magnetic resonance imaging (hyperpolarized MRI), it may be possible to further improve diagnostic correctness and precision as well as guide personalized treatment planning. Ultimately, incorporating these advanced approaches can significantly advance the field of personalized oncology.

Taken together, future advances in cancer therapy will depend on a deeper understanding of metabolic reprogramming, tumor-type-specific metabolic characteristics, and the influence of microenvironmental crosstalk. Equally important will be the application of advanced diagnostic technologies to assess metabolic heterogeneity. Therapeutic progress will also require the integration of metabolic modulators with standard treatments and dietary interventions as well as exploration of the complex interactions between nutrition, the microbiome, and treatment responses. Finally, incorporating metabolomic analyses and functional imaging into clinical workflows may further enhance the precision and effectiveness of personalized oncology.

## Figures and Tables

**Table 2:** Mapping the metabolic landscape of cancer: therapeutic interventions targeting glucose (part A), lipid (part B), and amino acid (part C) metabolism.

**Part A**							
**Target/Enzyme/Transporter**	**Inhibitor/Compound**		**Associated Cancer Types**			**Clinical Trial Phase/Status**	
Hexokinase (HK)	2-Deoxy-D-glucose (2-DG)		Leukemia (P388), solid tumors			Phase I (NCT00096707), limited efficacy	
GLUT1/GLUT family	Under investigation		Various solid tumors			Preclinical/exploratory	
Sodium-Glucose Cotransporter2 (SGLT2)	SGLT2 inhibitors (e.g., dapagliflozin, empagliflozin)		Hepatocellular carcinoma (HCC), T2D-associated cancers			Observational/real-world studies; no defined clinical trial phase	
Pyruvate Kinase M2 (PKM2)	Not specified		Lung adenocarcinoma, triple-negative breast cancer (TNBC)			Preclinical; prognostic marker	
Lactate Dehydrogenase A (LDHA)	FX11, oxamate, dichloroacetate, PSTMB		Neuroblastoma, breast (MCF-7), liver (Hep3B), colon (HT29), others cancers			Preclinical	
Monocarboxylate Transporter 1 (MCT1)	AZD3965		Lung, breast, metabolic symbiotic tumors			Phase I/II (NCT01791595)	
Monocarboxylate Transporter 4 (MCT4)	Under investigation		Hypoxic/metastatic tumors			Preclinical	
Pyruvate Dehydrogenase (PDH)	Devimistat (CPI-613)		Acute myeloid leukemia (AML), pancreatic cancer			Phase III (NCT03504410, failed)	
α-Ketoglutarate Dehydrogenase (KGDH)	Devimistat		AML, pancreatic adenocarcinoma			Phase III (AVENGER 500, no clinical benefit)	
Pyruvate Carboxylase (PC)	Not specified		Metastatic breast cancer			Preclinical; linked to metastasis	
Acetyl-CoA Carboxylase 1 (ACC1)	Not specified		Breast cancer (mesenchymal phenotype)			Preclinical; epigenetic target	
Proton pumps/Tumor pH regulation	TRIS buffer (Tris–base)		Various solid tumors			Experimental; limited clinical feasibility	
Lactate (as a target)	Alkaline buffers (e.g., TRIS), MCT inhibition		Multiple cancer types			Preclinical/early clinical	
**Part B**							
**Target/Enzyme/Transporter**	**Function/Mechanism**	**Associated Cancer Types**		**Inhibitor/Compound**			**Clinical Status/Notes**
Fatty Acid Synthase (FASN)	De novo fatty acid synthesis (palmitate)	Breast (esp. TNBC), lung, prostate, colorectal, ovarian cancers		TVB-2640 (denifanstat), Orlistat, PPIs			Multiple Phase I/II trials (NCT02223247, NCT03808558); sensitizes to chemo/radiotherapy
ATP-Citrate Lyase (ACLY)	Citrate → Acetyl-CoA, key in lipogenesis	Breast, prostate, pancreatic cancers		Bempedoic acid (ETC-1002)			Preclinical/early clinical; sensitizes prostate CA to AR antagonism
Acetyl-CoA Carboxylase (ACC)	Acetyl-CoA → Malonyl-CoA (rate-limiting step in FA synthesis)	Breast cancer (mesenchymal subtypes)		ND (not detailed)			Preclinical
Acetyl-CoA Synthetase 2 (ACSS2)	Acetate → Acetyl-CoA (supports lipid synthesis under stress)	Various tumors under hypoxia		ND			Preclinical
Stearoyl-CoA Desaturase (SCD)	Converts saturated FA to MUFA (for membrane fluidity, signaling)	Breast, thyroid cancer and others		SCD inhibitors (unspecified)			Preclinical; silencing inhibits proliferation/migration
Fatty Acid Desaturase 2 (FADS2)	Palmitate → sapienate (alternative desaturation)	SCD-resistant tumors (HCC, lung)		ND			Dual inhibition (SCD + FADS2) shows tumor reduction
Fatty Acid Transporters (FATPs)	Long-chain FA uptake	Breast, ovarian, metastatic tumors		ND			Preclinical; key in FA scavenging from microenvironment
CD36 (Fatty Acid Translocase)	FA uptake + signaling receptor	Breast, ovarian (esp. metastatic)		Anti-CD36 antibodies (experimental)			Target for blocking metastasis
Fatty Acid Binding Proteins (FABP4/5)	Intracellular FA transport	Breast cancer, macrophages, adipocytes in TME		FABP4/5 inhibitors (unspecified)			Preclinical; prognostic markers; hypoxia-induced expression
Carnitine Palmitoyltransferase 1 (CPT1A)	Controls FA entry into mitochondria for β-oxidation	Various solid tumors (esp. metastatic)		Etomoxir (experimental)			Preclinical; associated with resistance to metabolic stress; granted orphan drug status for malignant glioma
Acyl-CoA Dehydrogenases	Initiates fatty acid β-oxidation	Not specified		ND			Preclinical
PPARs (esp. PPARα/δ)	Regulate FAO, lipid metabolism, and immune modulation	Breast, prostate, colorectal cancers		Agonists/antagonists			Experimental; role in immune evasion and metabolic flexibility
Lipid Droplets (LD) Formation	Store excess FA; source of NADPH and energy	Breast, prostate, metastatic cancers		ND			Protective against lipotoxicity and ROS; linked to aggressiveness
Oncogenic Signaling & Lipid Metabolism	EGFR/HER1/2 → PI3K-Akt-mTOR → FASN upregulation	Breast (esp. HER2+), lung (KRAS-mutant)		Indirect targeting via FASN/PI3K/mTOR inhibitors			Supports autocrine loop; contributes to resistance and metastasis
ROS Resistance via Lipid Remodeling	FA saturation status alters membrane permeability/resistance	Castration-resistant prostate cancer		ND			Lipidomic signatures correlate with poor prognosis
FA Metabolism in Immune Evasion	Alters T cell and dendritic cell function in TME	Solid tumors		Lipid metabolism modulators (e.g., FASN inhibitors)			Enhances efficacy of immunotherapy (checkpoint blockade etc.)
**Part C**							
**Tatget/Enzyme/Transporter**	**Inhibitor/Compound**	**Associated Cancer Types**			**Clinical Trial Phase/Status**		
Glutaminase (GLS1)	Telaglenastat (CB-839)	Triple-negative breast cancer, hematological malignance, glioblastoma, renal carcinoma, colorectal, lung and cervical cancers			Phase I—completed in hematologic cancers (NCT02071862) Phase I/II + nivolumab—not demonstrated therapeutic efficacy in advanced solid tumors (NCT02861300) Phase Ib/II + talazoparib—completed in solid tumors (NCT03875313) Phase II in NSCLC—terminated (NCT02771626) Phase II in RCC + everolimus—completed (NCT03163667) Phase II in RCC + cabozantinib—did not meet primary endpoint (NCT03428217) Fast Track status + cabozantinib in metastatic RCC—currently ongoing		
Glutaminase (GLS1)	IPN60090 (IACS-6274)	Advanced solid tumors			Phase I in advanced solid tumors—completed (NCT04771783) Phase I + pembrolizumab, paclitaxel, bevacizumab—ongoing (NCT05340835)		
Glutaminase (GLS1)	bis-2-(5-phenylacetamido-1,2,4-thiadiazol-2-yl)ethyl sulfide (BPTES)	Pancreatic, ovarian cancers, alveolar adenocarcinoma, non-small cell lung cancer, hepatocellular carcinoma			Preclinical		
Glutaminase C (GAC)	UPGL00004	Triple-negative breast cancer (TNBC) cells			Preclinical		
Glutamine transporter ASCT2 (SLC1A5)	V-9302	Renal carcinoma, breast, colon cancers			Preclinical		
Glutamine transporter ASCT2 (SLC1A5)	MEDI7247 (antibody)	Hematological malignancies, advance or metastatic solid tumors			Phase I (NCT03811652, NCT03106428)—completed		
Glutamine transporter ASCT2 (SLC1A5)	L-γ-Glutamyl-p-nitroanilide (GPNA)	Lung, stomach, colon and prostate cancers			Preclinical		
Glutamine antagonist	6-Diazo-5-oxo-L-norleucine (DON)	Glioblastoma, pancreatic cancer, sarcomas, leukemias, medulloblastoma			Phase I/II—halted due to toxicity and discontinued		
Glutamine antagonist	JHU-083 (pro-drug of DON)	Prostate, bladder and colon cancers, lymphoma, melanoma, medulloblastoma			Preclinical		
Glutamine antagonist	Sirpiglenastat (pro-drug of DRP-104)	Advanced solid tumor			Phase I/II—ongoing (NCT04265534);		
Glutamine amidotransferases/Glutamine antagonist	azaserine	Pancreatic and colorectal cancers, solid tumors, breast cancer, sarcoma			In solid tumor—halted due to toxicity, preclinical		
γ-Glutamyl transferase (γ-GT)/Glutamine analog	Acivicin	Leukemia, breast cancer, and ovarian carcinoma, lung cancer			Phase II; halted due to toxicity		
3-phosphoglycerate dehydrogenase (PHGDH)	NCT-502/NCT-503	Breast cancer, melanoma and cervical cancer			Preclinical		
3-phosphoglycerate dehydrogenase (PHGDH)	PKUMDL-WQ-2101/-2201	Breast cancer			Preclinical		
3-phosphoglycerate dehydrogenase (PHGDH)	CBR-5884	Ovarian and breast cancers			Prelinical		
3-phosphoglycerate dehydrogenase (PHGDH)	BI-4916	Cervical, breast cancer and acute myeloid leukemia			Preclinical		
Serine hydroxymethyltransferase 1 and 2 (SHMT1/2)	sertraline (antidepressant repurposed)	Breast cancer, lung cancer, glioblastoma, leukemia			Phase I + cytosine Arabinoside in leukemia (NCT02891278)—completed Phase II + metronomic temozolomide in glioblastoma (NCT02770378)—completed Phase II in breast cancer (NCT00667121) completed		
L-type amino acid transporter 1 (SLC7A5)	JPH203 (KYT-0353)	Solid tumors			Phase I (NCT02028403)—completed Phase I/II ongoing (NCT04671432)		
Branched-Chain Aminotransferase 1 (BCAT1)	ERG240	Lung cancer			preclinical		
Branched-Chain Ketoacid Dehydrogenase Kinase (BCKDK)	BT2 (3,6-dichlorobenzo[b]thiophene-2-carboxylic acid)	Lung cancer, triple-negative breast cancer, colorectal cancer			Preclinical		
Arginase 1/2 (ARG1/2)	CB-1158 (INCB001158)	Solid tumor			Phase I/II ongoing (NCT02903914)		
Arginase (cobalt-substituted human arginase)	HuArgI (Co)-PEG5000 (Pegylated cobalt-substituted human arginase)	Hepatocellular carcinoma, melanoma			Preclinical		
Arginine Deiminase (enzyme degrading arginine)	ADI-PEG20 (Pegylated Arginine Deiminase)	Hepatocellular carcinoma, mesothelioma and metastatic melanoma			Phase I ongoing (NCT02732184) Phase I/II completed (NCT02353318, NCT03455140, NCT01092091, NCT00988195, NCT02285101) Phase III (NCT01287585) in hepatocellular carcinoma—failed		
Arginase (recombinant human arginase)	PEG-BCT-100 (Pegylated recombinant human arginase)	solid tumors					
xCT (SLC7A11/SLC3A2; cystine/glutamate antiporter)	Erastin, imidazole ketone erastin	Solid tumors			Preclinical		
xCT (SLC7A11 cystine/glutamate antiporter)	Sulfasalazine	Glioma, pancreatic, lung and colorectal cancers, leukemia			Phase I/II in gliomas (ISRCTN45828668)—failed		
Glutamate-cysteine ligase (GCL)	Buthionine sulfoximine (BSO)	Multiple cancer types			Phase I in neuroblastoma—completed		
Isocitrate dehydrogenase 1 (IDH1)	Ivosidenib (AG-120)	AML (mut. IDH1), cholangiocarcinoma (IDH1 mut.), glioma			Phase I in solid tumor, including glioma (IDH1-mutant) (NCT02073994) Phase Ib in Relapsed/Refractory MDS (IDH1-mutant)—FDA approved October 2023 (NCT02074839) Phase Ib/II in newly diagnosed AML (IDH1-mutant in patient who are not candidates to receive intensive induction chemotherapy, FDA approved July 2022 (NCT02677922) Phase III + azacitidine in AML with an IDH1 Mutation, FDA approved July 2022 (AGILE trial) (NCT03173248) Phase III in Cholangiocarcinoma (advanced/metastatic, IDH1-mutant) FDA approved August 2021 (NCT02989857)		

## Abbreviations

The following abbreviations are used in this manuscript:

a.a.	amino acid
ACC	Acetyl-CoA carboxylase
ACC1	Acetyl-CoA carboxylase 1
ACLY	ATP-citrate lyase
ACSS2	Acetyl-CoA synthetase 2
α-KG	Alpha-ketoglutarate
AML	Acute myeloid leukemia
AMPK	AMP-activated protein kinase
Arg	Arginine
ARG	Arginase
Asp	Aspartate
ASS1	Argininosuccinate synthase 1
BAK	Bcl-2 homologous antagonist/killer
BAX	Bcl-2-associated X protein
BCAA	Branched-chain amino acid
BCAT	Branched-chain amino acid transaminase
BCKDK	Branched-chain alpha-ketoacid dehydrogenase kinase
CAFs	Cancer-associated fibroblasts
CPT1	Carnitine palmitoyltransferase 1
DG	D-glucose
DHA	Docosahexaenoic acid (an omega-3 fatty acid)
EPA	Eicosapentaenoic acid (an omega-3 fatty acid)
FA	Fatty acid
FAO	Fatty acid oxidation
FASBP	Fatty acid binding protein
FASN	Fatty acid synthase
FATP	Fatty acid transporter protein
Gln	Glutamine
GLS	Glutaminase
Glu	Glutamate
GLUD	Glutamate dehydrogenase
GLUT	Glucose transporter
Gly	Glycine
GPX	Glutathione peroxidase
HIF1α	Hypoxia-inducible factor 1 alpha
HK	Hexokinase
HMG-CoA	3-Hydroxy-3-methylglutaryl-coenzyme A
IDH	Isocitrate dehydrogenase
KGDH	α-ketoglutarate dehydrogenase
LDHA	Lactate dehydrogenase A
MDS	Myelodysplastic syndrome
MCT	Monocarboxylate transporter
MMP	Matrix metalloproteinase
MRI	Magnetic resonance imaging
MRS	Magnetic resonance spectroscopy
MUFA	Monounsaturated fatty acid
NO	Nitric oxide
PC	Pyruvate carboxylase
PDH	Pyruvate dehydrogenase
PET	Positron emission tomography
PFK	Phosphofructokinase
PHGDH	Phosphoglycerate dehydrogenase
PI3K	Phosphoinositide 3-kinase
PK	Pyruvate kinase
PPAR	Peroxisome proliferator-activated receptor
PPI	Proton pump inhibitor
PUFA	Polyunsaturated fatty acid
RCC	Renal cell carcinoma
SCD	Stearoyl-CoA desaturase
Ser	Serine
SGLT	Sodium/glucose co-transporter
SHMT	Serine hydroxymethyltransferase
T2D	Type 2 diabetes mellitus
TCA	Tricarboxylic acid cycle (Krebs cycle)
TME	Tumor microenvironment
VEGF	Vascular endothelial growth factor
